# Regular moist snuff dipping does not affect endurance exercise performance

**DOI:** 10.1371/journal.pone.0181228

**Published:** 2017-07-13

**Authors:** Frida Björkman, Fredrik Edin, C. Mikael Mattsson, Filip Larsen, Björn Ekblom

**Affiliations:** 1 Åstrand Laboratory of Work Physiology, The Swedish School of Sport and Health Sciences, Stockholm, Sweden; 2 Department of Food and Nutrition, and Sport Science, University of Gothenburg, Gothenburg, Sweden; 3 Department of Physiology and Pharmacology, Karolinska Institute, Stockholm, Sweden; Telethon Institute for Child Health Research, AUSTRALIA

## Abstract

Physiological and medical effects of snuff have previously been obtained either in cross-sectional studies or after snuff administration to non-tobacco users. The effects of snuff cessation after several years of daily use are unknown. 24 participants with >2 years of daily snuff-use were tested before and after >6 weeks snuff cessation (SCG). A control group (CO) of 11 snuff users kept their normal habits. Resting heart rate (HR) and blood pressure (BP) were significantly lower in SCG after snuff cessation, and body mass was increased by 1.4 ± 1.7 kg. Total cholesterol increased from 4.12 ± 0.54 (95% CI 3.89–4.35) to 4.46 ± 0.70 (95% CI 4.16–4.75) mM L^–1^ in SCG, due to increased LDL, and this change was significantly different from CO. Resting values of HDL, C-reactive protein, and free fatty acids (FFA) remained unchanged in both groups. In SCG group, both HR and BP were reduced during a four-stage incremental cycling test (from 50 to 80% of VO_2_max) and a prolonged cycling test (60 min at 50% of VO_2_max). Oxygen uptake (VO_2_), respiratory exchange ratio, blood lactate (bLa) and blood glucose (bGlu) concentration, and rate of perceived exertion (RPE) were unchanged. In CO group, all measurements were unchanged. During the prolonged cycling test, FFA was reduced, but with no significant difference between groups. During the maximal treadmill running test peak values of VO_2_, pulmonary ventilation (V_E_), time to exhaustion and bLa were unchanged in both groups.

In conclusion, endurance exercise performance (VO_2_max and maximal endurance time) does not seem to be affected by prolonged snuff use, while effects on cardiovascular risk factors are contradictory. HR and BP during rest and submaximal exercise are reduced after cessation of regular use of snuff. Evidently, the long-time adrenergic stress on circulation is reversible.

## Introduction

Swedish moist snuff contains nicotine (approximately 8 mg nicotine per gram of snuff) and several thousands of other ingredients. Nicotine acts in the body by binding to nicotinic acetylcholine receptors (nAChRs) which are widely spread and present both in the central nervous system and in muscles. The binding will release neurotransmitters such as dopamine and acetylcholine, and chronic nicotine exposure will lead to a desensitization of the receptor but also an up-regulation of the amount of binding site [1]. The use of oral moist snuff (“snus”) is widespread among athletes in the Nordic countries, Switzerland, and in the U.S. Snuff users are often involved in team sports, for example ice hockey and baseball [2–4]. The psychological and medical effects of nicotine and tobacco use are far more investigated then exercise related questions. Amongst several relevant exercise physiological effects nicotine increases the release of epinephrine and norepinephrine from the adrenal medulla and elevates blood pressure (BP) and heart rate (HR) both at rest and during submaximal exercise [5–8]. Snuff has a carcinogenic effect on some organs [9, 10], and previous studies have shown an increased all-cause mortality in snuff users [11, 12]. The effect on cardiovascular disease (CVD) risk factors is unsettled. Some studies report no differences between regular snuff users and non-users [13, 14], while others have shown negative effects on blood lipid profile in snuff using baseball players [15]. The risk of myocardial infarction is debated with studies showing for [11, 16] and against [17], the latter supported by a recent review [18]. Furthermore, a two-year follow-up study has shown that snuff cessation after a myocardial infarction decreased the mortality rate with almost 50% [19].

The effect of snuff on endurance exercise performance is unsettled. Bolinder et al. [20] found no differences in peak oxygen uptake (VO_2_peak) in well-trained snuff users compared to non-users. Nor did Bahrke et al. [21] or Baldini et al. [22] find any difference between non- and regular snuff users regarding exercise performance, while Mündel & Jones [23] reported increased endurance during submaximal cycling after administration of nicotine. Exposure to snuff may [24] or may not [23, 25] increase blood lactate concentration (bLa) during submaximal exercise.

However, all these results have been obtained in either cross-sectional studies comparing snuff and non-tobacco users or after acute administration of nicotine or snuff to non-tobacco users. Since Bahrke et al. [21] found a negative relation between years of snuff use and endurance exercise performance we hypothesised that the long-term adrenergic stimulus of nicotine would have a negative effect on a) endurance exercise performance, b) the acute circulatory and metabolic adaptation to submaximal and maximal exercise and c) some important CVD risk factors, but furthermore that these negative effects would be reversed with cessation of snuff (nicotine) use.

## Material and methods

Forty-two regular snuff users were recruited to the study. Of them, the 30 that stated that they were motivated to try to abstain from snuff for a longer period were allocated in the snuff cessation group (SCG) and the remaining 12 constituted the control group (CO). Inclusion criteria for the study were >2 years of daily use of Swedish snuff (*i*.*e*. oral moist snuff, “snus”), no illnesses or medications and regular physical activity habits (*i*.*e*. strenuous endurance exercise >3 time a week). Exclusion criteria were cigarette smoking and known cardiovascular or other diseases. In the SCG two participants reported that they could not abstain from using snuff and they terminated their participation after the first test session. A total of three participants (two in SCG and one in CO) had to withdraw from the study due to different injuries. Finally, two participants in SCG were later excluded due to elevated cotinine values (>10 ng mL^–1^) in the post SC test. Cotinine is the major metabolite of nicotine, and therefore a suitable biomarker for nicotine exposure both in smokers and non-smoking tobacco users [26]. Subject characteristics for the 24 participants in the SCG and the 11 in the CO finally included in the study are given in Table 1. Because of some missing data the *n*-numbers may vary in the final analyses and presentation of the results.

**Table 1:** Participant characteristics.

	SCG *(n = 24)*	CO *(n = 11)*	p =
Age (years)	33.0 ± 7.2	38.6 ± 6.7	*
Height (cm)	177.4 ± 9.3	178.6 ± 10.6	ns
Body mass (kg)	76.7 ± 10.5	75.1 ± 12.5	ns
BMI (kg/m^2^)	24.3 ± 2.5	23.2 ± 3.0	ns
Start of regular snuff use (years of age)	18.9 ± 4.8	18.7 ± 3.7	ns
Length of snuff use (years)	14.1 ± 6.7	19.8 ± 6.7	*
Average consumption (45 g boxes per week)	4.1 ± 1.7	4.4 ± 2.0	
VO_2_max familiarization-test (mL kg ^–1^ min ^–1^)	51.6 ± 7.6	51.5 ± 5.4	

Data are presented as arithmetic mean ± SD.

SCG, snuff cessation group (5 females, 19 males); CO, control group (2 females, 9 males).

* p<0.05 significant difference between SCG and CO.

** p<0.01 significant difference between SCG and CO.

ns = no significant difference.

All participants were fully informed about the procedures and their right to terminate the experiment at any point. Written informed consent was obtained. The design of the study was conformed to the Declaration of Helsinki and approved by the Regional Ethics Committee in Stockholm, ref. no. 2009/829-31/3.

### Familiarization test

During the weeks before the main experiment started all participants visited the laboratory in order to get acquainted with all test methods used. They also performed an individually designed maximal running test (RUN_max_) until voluntarily exhaustion. The individualized protocol was done with increments every minute, with the aim of passing the anaerobic threshold (RER >1.0) after approximately 3–4 min and with aimed total test time of 6–10 min. The test was performed on a treadmill (Rodby Electronics, Vansbro, Sweden) for determination of peak values on endurance time, maximal oxygen uptake (VO_2_max), HR, pulmonary ventilation (V_E_) and bLa. VO_2_max was reached according to Åstrand & Rodahl [27] when *1*) levelling off of VO_2_ *vs*. rate of work with VO_2_ on the highest work rate being within 150 mL min^-1^ from previous highest obtained value in the test, *2*) total work time was >5 min, *3*) bLa >9 mM L^-1^ and 4) subjective rating of perceived exertion (RPE) >16 according to Borg’s 6–20 RPE scale [28]. VO_2_ was analysed using an automatic metabolic system (Jaeger Oxycon Pro, Erich Jaeger, Germany). Before each VO_2_ measurement ambient temperature, humidity and barometric pressure were measured. The gas analyser was calibrated against a high precision gas mixture (16.00 ± 0.04% O_2_ and 4.00 ± 0.1% CO_2_, Air Liquide, Kungsängen, Sweden). Capillary blood samples were collected from the fingertip within 1 min and 3 min after the end of RUN_max_ for later analyses of bLa and bGlu (Biosen C-Line Sport, EKF Diagnostics, Magdeburg, Germany). RPE according to the Borg scale [28] modified by Ekblom and Goldbarg [29] for breathing and leg muscle fatigue, respectively, were obtained after each exercise test. HR during exercise tests was measured using the HR-monitor S610i (Polar Electro Oy, Kempele, Finland).

### Pre SC tests

The participants were instructed to keep the exact same diet the 24 hours prior to the pre- and post-tests, and during these periods they were also instructed to refrain from strenuous exercise and all types of stimulants (*e*.*g*. drugs, alcohol, coffee, tea). At the Pre SC test day they arrived in the laboratory >3 hours after a light meal. Two hours and also 30 min before arrival they were asked to take in their normal dose of snuff which was kept in the mouth until the rest measurements started. Dosage may differ between subjects due to compound of moist snuff, see also “average consumption” (Table 1). Body mass was obtained on a standard controlled scale to the nearest 0.1 kg. After 15 min of supine rest the systolic and diastolic BP was measured using sphygmomanometer and stethoscope. Mean BP was calculated as diastolic BP + 1/3 of the pulse pressure (difference between systolic and diastolic BP). A venous blood sample for later analyses of different parameters (see below) was obtained.

Thereafter the participants carried out a four-stage (à 5 min) intermittent incremental cycling test (IIC-test) with 1 min rest between stages in order to establish the circulatory adaptation to submaximal exercise. Workloads were set to correspond to 50, 60, 70 and 80% of previously determined individual VO_2_max at treadmill running. The test was carried out on a cycle ergometer (Monark ergomedic 839 E, Monark, Sweden) with pedal frequency 70 rpm. Ventilatory measurements (VO_2_, VCO_2_, V_E_), HR, BP, bLa and RPE were recorded during the last minute of each 5 min stage. A capillary blood sample for analyses of bLa and bGlu was collected from the fingertip during the 1 min rest between each workload. After 10 min of rest and a short warm-up jog the standardized RUN_max_ was carried out in the same way as described above in order to establish VO_2_max, endurance time and the cardiovascular and metabolic responses to maximal exercise.

After RUN_max_ the participants were given 200 ml of 5% glucose in water, a venous catheter was inserted in a superficial elbow vein, and they were rested for 60 min. After 30 min of this rest they took in another dose of their normal amount of snuff and held it until the start of the prolonged 60 min cycling test (60 min test) where they cycled on the Monark ergometer at a workload corresponding to 50% of individual VO_2_max. At the 5^th^, 30^th^ and 55^th^ min HR and ventilatory measurements were recorded for five minutes. Respiratory exchange ratio (RER) was calculated from VCO_2_ and VO_2_. At these time points a blood sample for determination of FFA, bLa and bGlu was obtained, and measurements of BP and RPE were done. This test was carried out in order to analyse the cardiovascular and metabolic adaptation to prolonged exercise of moderate intensity.

**Fig 1:**
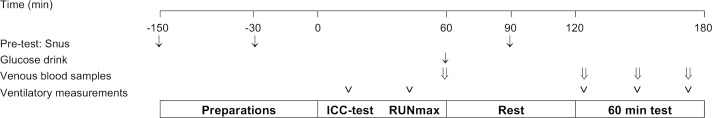
Schematic overview of the experimental protocol. The procedures were identical pre- and post SC, except from the intake of snuff, which was absent in the post SC tests.

A schematic overview of the experimental protocol is shown in Fig 1. After the end of these pre SC-tests the participants in the SCG were instructed to abstain from any tobacco or nicotine use and also to keep their normal physical activity and diet habits during the coming SC period (SCP), during which they were regularly contacted for support and were interviewed regarding their compliance to abstain from snuff.

### Post SC tests

When choosing the duration of the cessation period the aim was to have enough time to normalise biochemical alteration due to nicotine exposure, but not so long that the physical status could risk to be changed markedly due to other reasons then withdrawal from nicotine. According to a study by Cosgrove and colleagues [30], the availability of β2*-nAChR (neuronal subunit) in tobacco smokers was higher compared to controls one week after cessation, but there was no difference at the measurements 4, 6 or 12 weeks after cessation. Their conclusion was that higher receptor availability can persist for up to four weeks but is normalized to the level of non-smokers after 6 weeks. Thus, the post SC tests were performed >6 weeks after the pre-test. This holds true for all subjects except two participants in CO who due to logistics had to perform their post-test just short of 6 weeks after pre-test. All tests were repeated in exactly the same way and time of day as during the pre-test, however, for the SCG all tests were obviously performed without snuff intakes before and during the test.

### Analyses of blood samples

Capillary blood samples were drawn from the fingertip, and analyses of bLa and bGlu were made with Biosen C-Line Sport (EKF Diagnostics, Magdeburg, Germany). In the venous blood samples, concentrations of cotinine and insulin were determined with commercially available ELISA kits (Cotinine, Bio-Quant Diagnostics, San Diego, USA; Insulin, Mercodia AB, Uppsala, Sweden). Furthermore, HDL, LDL, total cholesterol and C-reactive protein were analysed using absorbance measurements (Synchron LX® Systems, Beckman Coulter, Inc., USA) by the Karolinska Hospital Laboratory for Blood Chemistry (Stockholm, Sweden). FFA was enzymatically analysed using a reagent kit (Wako Chemicals GmbH, Neuss, Germany).

### Statistics

All statistical analysis was performed using Statistica 9 (StatSoft Ink., Tulsa, OK, USA).

Data were accepted for normal distribution using the Shapiro-Wilks W-test before performing parametric statistics. Student’s independent t test was used to evaluate differences between groups in descriptive variables. Correlations between cotinine levels and cholesterol levels pre SC and changes in HR were assessed using Pearson correlation coefficients (r). The multiple parameters over time during the IIC-test and 60 min submaximal test were analysed with a repeated measures ANOVA (RM ANOVA) with experimental group as the grouping factor and change (i.e. difference between pre- to post-test values) in each time point of sampling as the repeated measures. When a significant effect was found a post hoc analysis was done using the Fisher least significant difference post hoc test. These data are presented as means and 95% confidence intervals (CI). Additional data are presented as means ± SD, in relevant cases including range (min–max). Non parametric data from RPE ratings are presented as median (25^th^–75^th^ percentile). Significance was accepted at *p* < 0.05, and trends were considered at 0.05 < *p* < 0.1.

## Results

### Rest

All values from the measurements and blood samples drawn during the first 15 min of rest are presented in Table 2. Body mass increased significantly during the SC period (SCG 1.4 ± 1.7 kg and CO 0.5 ± 1.1 kg) but the increase was not statistically different between groups. Resting HR decreased by 5 ± 7 beats min^–1^ in the SCG, significantly different from values in CO (*p* = 0.001). Analysis of BP data revealed slightly lower post-values, but there were no statistical differences between groups.

**Table 2:** Resting values.

	SCG *(n = 24)*		CO *(n = 11)*				
	Pre SC	Post SC	Pre	Post	Group effect	Time effect	Interaction effect
Days between tests		48 ± 12		44 ± 9			
Cotinine (ng mL^–1^)	302 ± 119	n.d.	360 ± 102	381 ± 96	0.000*	0.000*	0.000*
Body mass (kg)	76.7 ± 10.5	78.0 ± 10.8	75.0 ± 12.6	75.6 ± 12.8	ns	0.003*	ns
BMI (kg/m^2^)	24.3 ± 2.5	24.8 ± 2.4	23.2 ± 3.0	23.4 ± 3.1	ns	0.003*	ns
Systolic blood pressure (mmHg)	126 ± 8	124 ± 8	124 ± 10	126 ± 11	ns	ns	ns
Diastolic blood pressure (mmHg)	70 ± 11	67 ± 10	74 ± 10	68 ± 8	ns	0.005*	ns
Mean blood pressure	89 ± 9	86 ± 8	90 ± 10	87 ± 8	ns	0.008*	ns
Heart rate (beats min^–1^)	61 ± 9	56 ± 8	59 ± 4	64 ± 11	ns	ns	0.001*
Blood lactate (mM L^–1^)	1.09 ± 0.33	1.00 ± 0.30	1.07 ± 0.26	1.06 ± 0.30	ns	ns	ns
Blood glucose (mM L^–1^)	4.93 ± 0.49	5.03 ± 0.49	5.11 ± 0.74	5.15 ± 0.71	ns	ns	ns
Total cholesterol (mM L^–1^)	4.12 ± 0.54	4.46 ± 0.70	4.64 ± 0.84	4.56 ± 0.64	ns	ns	0.073
LDL (mM L^–1^)	2.37 ± 0.43	2.68 ± 0.52	2.68 ± 0.80	2.57 ± 0.68	ns	ns	ns
HDL (mM L^–1^)	1.34 ± 0.29	1.35 ± 0.29	1.47 ± 0.43	1.49 ± 0.41	ns	ns	0.029*
FFA (mM L^–1^)	0.31 ± 0.25	0.25 ± 0.14	0.20 ± 0.11	0.24 ± 0.26	ns	ns	Ns
C-reactive protein (mg L^–1^)	1.5 ± 0.6	1.4 ± 0.5	1.0 ± 0.0	1.5 ± 0.7	ns	ns	ns
Insulin (mU L^–1^) *SCG n = 11*, *CO n = 10*	46.6 ± 41.2	40.5 ± 35.0	56.9 ± 37.8	98.0 ± 67.1	0.093	0.023*	0.003*

Data are presented as arithmetic mean ± SD.

SCG, snuff cessation group; CO, control group; n.d. = not detectable.

* Significant difference (p<0.05) from RM ANOVA.

ns = no significant difference

In the SCG total cholesterol increased by 0.34 ± 0.63 mM L^–1^ post SCP and there was a tendency towards a significant difference between SCG and CO (CO -0.08 ± 0.50, *p* = 0.07). LDL increased by 0.31 ± 0.54 mM L^–1^ in the SCG and there was a significant difference between the groups (CO -0.11 ± 0.31, *p =* 0.03). HDL, C-reactive protein, FFA, bLa and bGlu were unchanged after SCP. Insulin values were altered between tests, as evident by an interaction and time effect from a RM ANOVA. Post-hoc analysis revealed significantly higher post-test insulin in CO and the reason for that result was one individual with an extremely high post SC value.

In the SCG there was a significant negative correlation between the pre SCP cotinine value and the magnitude of the reduction in resting HR (r = -0.48, *p* = 0.02). There were also positive correlations between pre cotinine values and total cholesterol (r = 0.39, *p* = 0.02) and LDL (r = 0.36, *p* = 0.04). There were no correlations between pre-cotinine values and any other pre-values. There were no other correlations in pre-cotinine values and pre- to post differences in any measurements after the SCP.

### Submaximal exercise

During the IIC-test VO_2_, V_E_, RER and RPE were unchanged comparing pre- to post-values in both groups. Mean HR was 4.5 beats min^–1^ lower in the SCG post SC but the decrease was not statistically different from CO. RM ANOVA analysis of BP data revealed no interaction effect between the SCG and CO. The decrease in systolic BP in SCG where significantly greater than in CO, as evident by an overall significance for group (SCG -14, 95% CI -18 to 9 and CO 0.8, 95% CI -6 to 8 mmHg, respectively). Diastolic BP where unchanged and there was no difference between groups, but there was a tendency towards a significant difference between groups regarding mean BP (*p* = 0.08).

**Fig 2:**
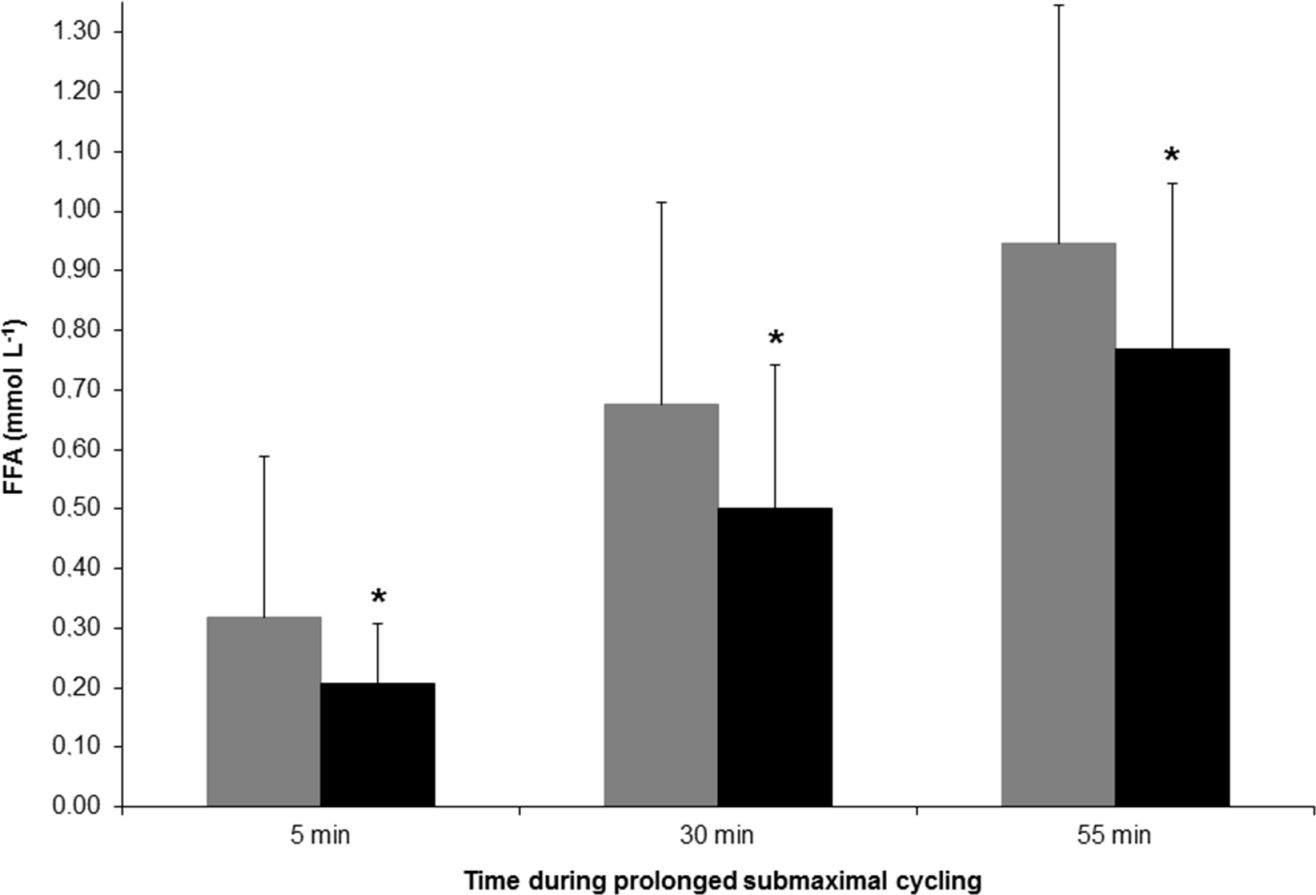
Plasma levels of free fatty acids (FFA) at three time points during the 60 min cycling exercise pre- (gray bars) and post- (black bars) snuff cessation (SC).

**Fig 3:**
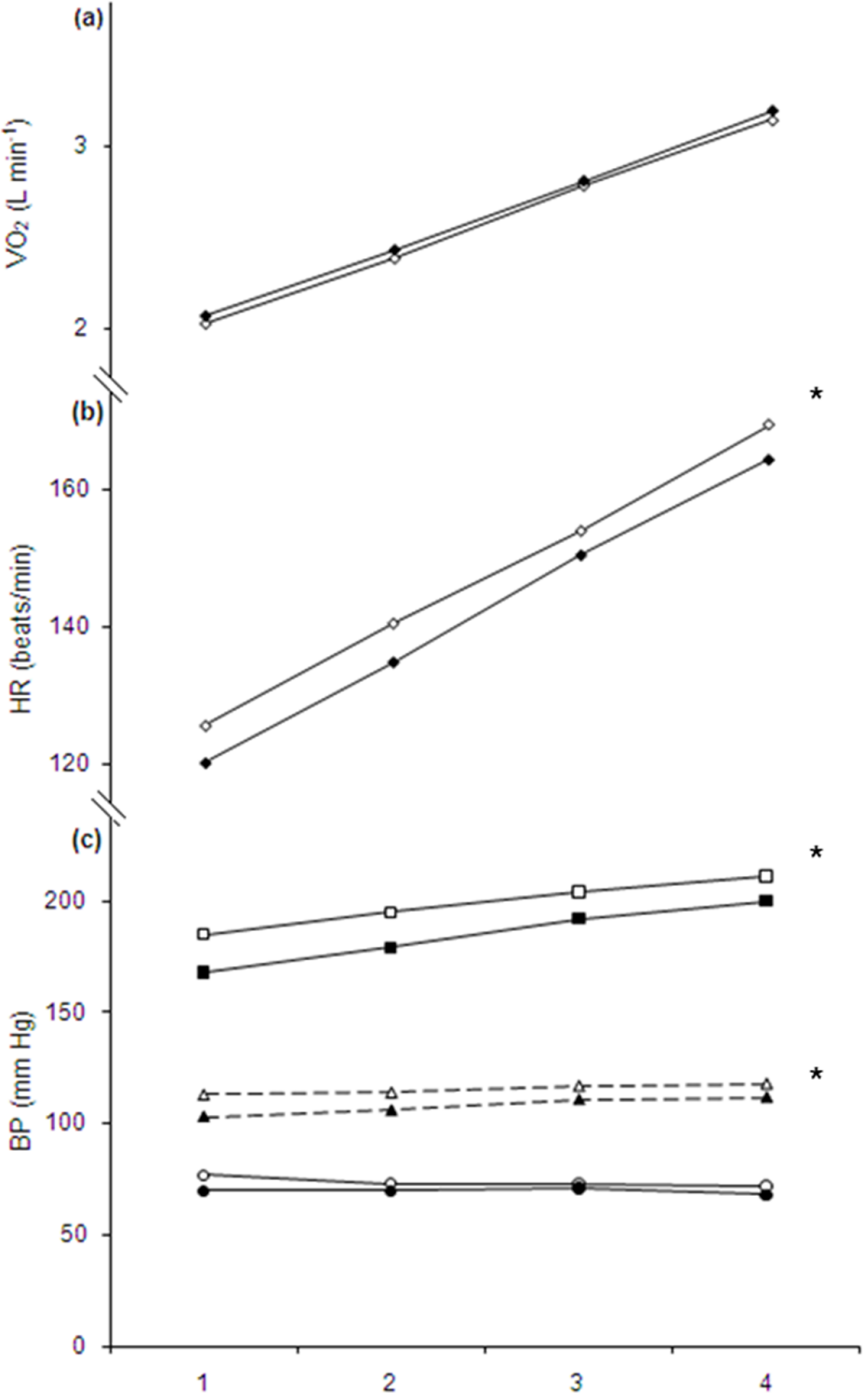
Mean changes in (a) oxygen uptake (VO_2_), (b) heart rate (HR), and (c) blood pressure (BP) during submaximal work rate 1: 50%, 2: 60%, 3: 70% and 4: 80% of individual VO_2_max. Open symbols indicate pre- and solid symbols indicate post-SCP (snuff cessation period). There were significant differences in mean value of HR and BP between pre- to post-SCP conditions (ANOVA). VO_2_ was unchanged.

During the 60 min test VO_2_, V_E_, bLa, RER, bGlu and RPE at the 5^th^, 30^th^ and the 55^th^ min time points were all unchanged after comparing corresponding time points before SCP. These values also remained unchanged in the CO. HR decreased in SCG (mean difference -4 ± 3 beats min^–1^) in accordance with the changes observed during the IIC-test, but the decreases were not significantly different from the values in CO. The change in systolic BP (SCG -6, 95% CI -11 to -0.3 and CO 7, 95% CI -0.8 to 16 mmHg), and mean BP (SCG -4, 95% CI -7 to -0.8 and CO 2, 95% CI -3 to 7) was significantly different between groups. Diastolic BP remained unchanged in SCG and CO. Mean FFA during the 60 min test was slightly lower in SCG during the post-test (mean difference -0.14 ± 0.25 mM L^–1^, see Fig 2 for details), but the change was not significantly different from the CO. The rate of increase in all the above mentioned parameters between the 5^th^ and the 55^th^ min time points were unchanged pre- to post-SCP, as evident by the absence of any interaction effect from a RM ANOVA. Values for VO_2_, HR and BP in the SCG during the 60 min test, pre- and post-SC, is shown in Fig 3.

### RUN_max_ test

**Fig 4:**
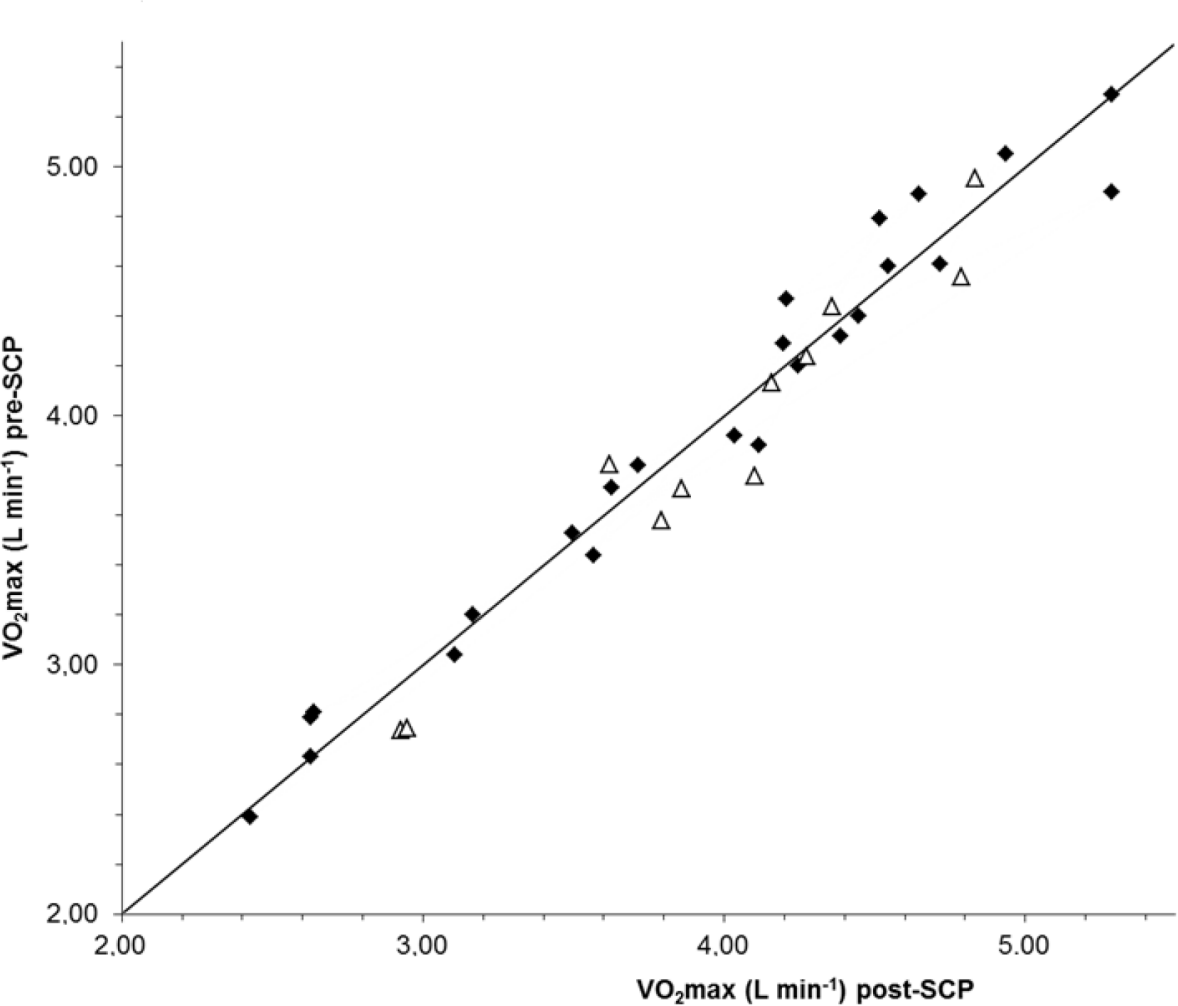
Individual VO_2_max pre- and post the snuff cessation (SC) or for the control situation. Solid symbols represent snuff cessation group and open triangles are the control group.

The VO_2_max in SCG was 3.94 ± 0.78 and 3.96 ± 0.77 L min^–1^, in the pre- and post-test respectively. The corresponding values in CO were 3.88 ± 0.70 and 3.97 ± 0.63 L^–1^, and there were no statistical differences between tests or between groups (Fig 4).

In the SCG mean time to exhaustion was 492 ± 61 and 501 ± 65 sec at the pre- and post SC test, respectively. The individually designed test protocols for RUN_max_ resulted in significantly shorter time to exhaustion in CO (382 ± 65 and 398 ± 54 sec, respectively), but there was no significant pre- to post-test difference within group.

For comparison, mean VO_2_max values in familiarization test and pre SC tests (n = 32) were almost identical: 3.94 ± 0.73 and 3.96 ± 0.73 L min^–1^. Time to exhaustion was 477 ± 79 and 461 ± 79 sec, respectively.

Pre- to post values of peak HR, V_E_, and RER and RPE were without statistical differences between groups or between tests in both groups. Peak concentrations of bLa and glucose were unchanged between tests. However, they were significantly lower in the CO compared to the SCG. All values are presented in Table 3.

**Table 3:** Peak values during maximal running tests in snuff cessation group (SCG) and control group (CO).

	SCG *(n = 24)*		CO *(n = 11)*				
	Pre SC	Post SC	Pre	Post	Group effect	Time effect	Interaction effect
VO_2_max (L min^–1^)	3.94 ± 0.78	3.96 ± 0.77	3.88 ± 0.70	3.97 ± 0.63	ns	0.084	ns
Time to exhaustion (sec)	492 ± 61	501 ± 65	382 ± 65	398 ± 54	0.000*	0.064	ns
HR_peak_ (beats min^–1^)	187 ± 9	188 ± 8	183 ± 8	184 ± 8	ns	ns	ns
V_E_ (L min^–1^)	146 ± 35	146 ± 35	151 ± 24	146 ± 23	ns	ns	ns
RER	1.11 ± 0.04	1.12 ± 0.04	1.12 ± 0.05	1.13 ± 0.05	ns	ns	ns
Blood lactate (mM L^–1^)	12.96 ± 2.56	13.16 ± 2.35	11.12 ± 3.14	10.65 ± 2.46	0.02*	ns	ns
Blood glucose (mM L^–1^)	6.89 ± 1.28	6.97 ± 1.25	5.54 ± 0.99	5.49 ± 0.83	0.02*	ns	ns
RPE (breathing)	19 (18–19)	18 (18–19)	19 (18–19)	19 (18–19)	ns	ns	ns
RPE (legs)	19 (18–19)	18 (18–19)	19 (18–20)	19 (19–20)	ns	ns	ns

Data are presented as arithmetic mean ± SD.

* Significant difference (p<0.05) from RM ANOVA. ns = no significant difference.

## Discussion

The aim of this study was to evaluate the effects of prolonged snuff use by studying long-established snuff dippers before and after a >6 weeks period of cessation of any tobacco use. Studies on receptor density [30] indicate that such a long period could be enough for normalizing possible effects of long-time use of snuff after the initial abstinence period. We hypothesized that in contrast to acute effects of snuff or nicotine administration the known enhanced sympathetic drive during long-time use of snuff would negatively affect endurance exercise performance. However, mean endurance time during the standardized RUN_max_ as well as mean VO_2_max were almost identical before and after the SCP with very narrow individual differences (Fig 4). The unchanged VO_2_max confirms the result from the cross-sectional study on Swedish snuff users compared to non-users [20]. On the other hand Mündel & Jones [23] reported an improved endurance during a submaximal time-trial cycling test after administration of nicotine, which, even though it is a different type of test, can be considered as a disagreement with our unchanged endurance time during maximal exercise. Endurance during submaximal exercise was not measured in this study, but bLa and RPE during both the four-stage IIC-test and the 60 min test were unchanged. The unchanged bLa in our study is in disagreement with the increased bLa in the cross-sectional placebo-controlled study of Van Duser & Raven [24] but agree with the results from Baldini et al. [25] and Mündel & Jones [23]. The unchanged RPE in our study is in agreement with data from both Perkins et al. [31] and Mündel & Jones [23]. Therefore, our conclusion is, in agreement with the results both from the cross-sectional study by Bolinder et al. [20] and single doses of snuff to non-tobacco users [21, 22], that endurance performance during dynamic exercise with large muscle groups involved is not influenced by long-time regular use of snuff as evaluated from >6 weeks cessation of snuff use.

The effects of snuff use on CVD risk factors are incoherent. To the best of our knowledge for the first time it can be settled that HR and BP both during rest and submaximal exercise are reduced after cessation of regular use of snuff. Evidently, the long-time adrenergic stress on circulation is reversible. Our results are in agreement with cross-sectional studies on snuff users or after acute administration of snuff or nicotine [5–8, 13]. We also found a positive relation between the pre-SCP cotinine and total cholesterol concentrations in agreement with results from Tucker et al. [32], which could indicate a negative effect of long-time use of snuff on total blood cholesterol metabolism. However, there was an increase in total cholesterol due to increased LDL cholesterol after SCP while HDL cholesterol remained unchanged. The reason for this increase in total cholesterol concentration, explained by enhanced LDL cholesterol, is unknown. However, one factor could be the increased body mass after SCP. Most participants reported that they had kept their physical activity patterns unchanged during the SCP, but many also reported that they had increased the intake of different types of snacks, such as chocolate and other fat rich products in order to dampen the nicotine abstinence feelings. The conclusion from this part of the present study is that the effects of snuff on CVD risk factors are unsettled. On the one hand an increased risk of regular snuff use is evident since both HR and BP at rest and during submaximal exercise and FFA during prolonged exercise are decreased after SCP and, furthermore, that there was a positive correlation between pre-SCP concentrations of cotinine and total cholesterol. But on the other hand both total and LDL cholesterol were increased after SCP. However, the latter effect on these blood lipids may be due to the increased body mass, indicating increased energy intake. Therefore, to evaluate the effect of snuff on different CVD risk factors further studies are essential, since both increased rates of myocardial infarctions [11, 16] and all-cause mortality [11, 12] are reported.

The strength of this study is the longitudinal design, the broad approach of investigations and well established laboratory methods. The cessation of snuff use was evaluated by regular contacts with the participants and a late measurement of cotinine in the end of SCP. The rest period between the RUN_max_ and the 60 min test could have been longer. However, for the analyses of the effect of cessation of snuff on metabolic adaptation to a prolonged submaximal exercise, evaluated at the 5^th^, 35^th^ and 55^th^ min time points, our impression is that the results are not influenced by this shortness since the same procedure was applied before and after SCP. There are also other limitations to the study, one being the lack of control of the participants’ daily life. They were all instructed to carry on with the same habits concerning physical activity, nutrition and diet during the entire time of the study, but this was not strictly controlled. Moreover, cotinine measurements were done only at the end of the SCP. However, based on the not-detectable values of cotinine in the post measurements (Table 2), and from our regular contacts with the 24 participants in the SCG, we are confident that they had abstained from snuff during the SCP. Another limitation is that the aim and relevant question is whether chronic snuff dipping impair endurance exercise performance and consequently the best way of investigating that would be to recruit participants measure their performance, randomize them in snuff-dipping group or controls and follow them for several years. We recognize that such a protocol would be interesting but also that it would be problematic both logistically and ethically, since tobacco use may induce several known medical risk factors. We believe that our protocol with long-term snuff dippers that quit their use might be the best possible way to address the situation. However, a follow-up study after 6 month, 1 year and/or even longer would give additional information.

## Conclusions

We conclude that long-time regular daily use of snuff does not affect endurance exercise performance in physical activities that engage large muscle groups. VO_2_max and time to exhaustion during a maximal running test were unchanged after compared to before the SCP. The effect of regular snuff use on CVD risk factors is incoherent, since some important risk factors (HR and BP) were improved after SCP while some other (total cholesterol, LDL and body mass) were negatively affected after snuff cessation.
